# Impact of Drug-Coated Balloon-Based Revascularization in Patients with Chronic Total Occlusions

**DOI:** 10.3390/jcm13123381

**Published:** 2024-06-09

**Authors:** Eun-Seok Shin, Ae-Young Her, Mi Hee Jang, Bitna Kim, Sunwon Kim, Houng Bang Liew

**Affiliations:** 1 Department of Cardiology, Ulsan University Hospital, University of Ulsan College of Medicine, Ulsan 44033, Republic of Korea; 0736353@uuh.ulsan.kr (M.H.J.); binna9044@gmail.com (B.K.); 2 Division of Cardiology, Department of Internal Medicine, Kangwon National University School of Medicine, Chuncheon 24289, Republic of Korea; 3 Department of Cardiology, Korea University Ansan Hospital, Ansan 15355, Republic of Korea; sunwon11@hanmail.net; 4 Cardiology Department and Clinical Research Center, Queen Elizabeth Hospital II, Kota Kinabalu 88300, Malaysia; hbliew22@gmail.com

**Keywords:** balloon angioplasty, drug-coated balloon, drug-eluting stent, coronary artery disease, percutaneous coronary intervention

## Abstract

**Background:** Percutaneous coronary intervention (PCI) with a drug-eluting stent (DES) for chronic total coronary occlusions (CTOs) improves clinical symptoms and quality of life. However, data on drug-coated balloon (DCB)-based PCI in CTO lesions are limited. **Methods:** A total of 200 patients were successfully treated for CTO lesions, either with DCB alone or in combination with DES (DCB-based PCI). They were compared with 661 patients who underwent second-generation DES implantation for CTO from the PTRG-DES registry (DES-only PCI). The endpoint was major adverse cardiovascular events (MACEs), which included a composite of cardiac death, myocardial infarction, stent or target lesion thrombosis, target vessel revascularization, and major bleeding at 2 years. **Results:** In the DCB-based PCI group, 49.0% of patients were treated with DCB only and 51.0% underwent the hybrid approach combining DCB with DES. Bailout stenting was performed in seven patients (3.5%). The DCB-based PCI group exhibited fewer stents (1.0; IQR: 0.0–1.0 and 2.0; IQR: 1.0–3.0, *p* < 0.001), shorter stent lengths (6.5 mm; IQR: 0.0–38.0 mm and 42.0 mm; IQR: 28.0–67.0 mm, *p* < 0.001), and lower usage of small stents with a diameter of 2.5 mm or less (9.8% and 36.5%, *p* < 0.001). Moreover, the DCB-based PCI group had a lower rate of MACEs than the DES-only PCI group (3.1% and 13.2%, *p* = 0.001) at 2-year follow-up. **Conclusions:** The DCB-based PCI approach significantly reduced the stent burden, particularly in the usage of small stent diameters, and resulted in a lower risk of MACEs compared to DES-only PCI in CTO lesions.

## 1. Introduction

Chronic total occlusion (CTO) of a coronary artery is relatively common, occurring in approximately 16% of patients diagnosed with coronary artery disease (CAD) [1]. Recent advancements in devices and treatment algorithms and growing experience have led to increased technical success in CTO percutaneous coronary intervention (PCI); however, the results regarding the clinical benefit of CTO remain inconclusive [2,3,4,5]. 

CTO PCI typically requires the use of multiple lengthy stents due to the lesion’s characteristics, leading to an increase in stent length. In the DECISION-CTO trial, an average of 2.3 stents were used, with a total length of 68.2 mm [4]. It is well known that a longer stent length independently predicts in-stent restenosis and stent thrombosis [6,7,8,9]. Furthermore, “full-metal jacket” PCI using overlapping drug-eluting stent (DES) is associated with a high rate of adverse events [10]. Moreover, stent implantation can hinder the restoration of vasomotion in stented segments and accelerate the development of neoatherosclerosis [11,12,13]. 

The use of drug-coated balloons (DCBs) in treating patients with in-stent restenosis [14] and de novo small vessel disease [15,16] has demonstrated comparable clinical outcomes to DES implantation. Unlike DES implantation, the DCB strategy leaves no material behind, thereby reducing the risk of stent-related adverse biological reactions that can lead to restenosis and thrombosis [17]. This approach promotes vascular healing. In particular, using DCB either alone or in combination with DES as a part of a hybrid procedure to decrease stent burden, especially in cases of small diameter stent implantation, could offer an alternative and beneficial treatment approach for CTO lesions. Recently, we reported that the DCB-based PCI approach, guided by the International Consensus Group [18] and Asian Pacific Consensus Group [19], significantly reduced stent burden for multivessel PCI, resulting in a lower rate of major adverse cardiovascular events (MACEs) at 2 years compared to DES-only PCI [20]. This study demonstrated the safe reduction in second-generation DES burden for lesions requiring multiple stents using the DCB-based PCI approach. However, the efficacy of DCB-based PCI for patients with CTO CAD has not been fully validated in the modern DES era. Hence, our aim was to evaluate the clinical significance of DCB-based PCI in patients with CTO CAD who underwent PCI with second-generation DES. 

## 2. Methods

### 2.1. Patient Population

A retrospective enrollment included a total of 200 patients who underwent successful PCI for CTO CAD using DCB alone or in combination with the DES hybrid approach. The patients were recruited from three university hospitals in South Korea (Ulsan University Hospital, Kangwon National University Hospital, and Korea University Ansan Hospital), all of which have experience in treating patients with CTO CAD using DCB (Impact of Drug-coated Balloon Treatment in de Novo Coronary Lesion; NCT04619277). 

We excluded patients meeting any of the following criteria from our analysis: a history of coronary artery bypass surgery, presentation with cardiogenic shock, administration of thrombolysis prior to PCI, suboptimal or failed PCI for target lesions, or loss to follow-up. Subsequently, we compared these findings with data from patients exclusively treated with DES for CTO CAD within the PTRG-DES (Platelet function and genoType-Related long-term ProGnosis in Drug-Eluting Stent-treated patients with coronary artery disease) consortium (ClinicalTrials.gov Identifier: NCT04734028) [21]. The study protocol received approval from the institutional review board of each participating center (approval code: 2021-07-036; approval date: 18 August 2021), and all patients provided written informed consent at the time of enrollment.

### 2.2. Procedure

All patients received 200 mg aspirin and 300 or 600 mg clopidogrel as loading doses before the procedure. Before proceeding with diagnostic coronary angiography, intracoronary nitroglycerin (200 μg) was administered to the patients. In CTO lesions, predilation balloon angioplasty was carried out to assess the feasibility of DCB treatment. For the DCB-based treatment group, interventions followed the recommendations outlined in international and Asia–Pacific consensus guidelines for DCB treatment [18,19]. All patients underwent predilation with either semi-compliant, non-compliant, or scoring balloons, adhering to the recommended balloon-to-vessel ratio of 0.8 to 1.0. Following predilation balloon angioplasty, stenting was postponed in cases of all types of dissections, as long as thrombolysis in myocardial infarction (TIMI) flow grade 3 was present. However, if predilation resulted in flow-limiting dissection (TIMI flow grade < 3), stent implantation was recommended without using a DCB. The DCB was inflated to its nominal pressure for a minimum of 60 s, ensuring that it extended at least 2 mm beyond the length of predilatation. Each DCB was coated with 3.0 μg/mm^2^ of paclitaxel combined with iopromide (SeQuent Please, B. Braun) as a carrier for the drug. Following the use of DCB, the final assessment was performed at least 5 min after administering a bolus of intracoronary vasodilator to prevent potential acute vessel closure. In cases with a high thrombus burden, a rescue strategy involving the use of glycoprotein IIb/IIIa receptor inhibitors was employed. The duration of the dual antiplatelet therapy was determined at the discretion of the attending physician.

### 2.3. Clinical Follow-Up and Endpoints

All 861 patients underwent clinical follow-up after the index procedure through outpatient clinic visits and telephone interviews. The study endpoint was the cumulative occurrence of MACEs, which included cardiac death, myocardial infarction, stroke, stent or target lesion thrombosis, target vessel revascularization, and major bleeding within a 2-year period. Cardiac death was defined as any death not distinctly attributable to non-cardiac causes, including myocardial infarction, according to previously published guidelines [11]. The definition of definite stent thrombosis adhered to the parameters outlined by the Academic Research Consortium definition [22], and major bleeding was defined as Bleeding Academic Research Consortium types 3 to 5 bleeding [23].

### 2.4. Statistical Analysis

Clinical characteristics were presented as percentages for categorical variables and as means with standard deviations or medians with interquartile ranges (Q1–Q3) for continuous variables. Group comparisons were performed using Pearson’s Chi-squared test or Fisher’s exact test for categorical variables and Student’s *t*-test or Mann–Whitney U-test for continuous variables, as appropriate. To compare clinical outcomes between the two groups, we evaluated the cumulative incidences of MACEs and other outcomes using the Kaplan–Meier method and then compared the curves using the log-rank test. To minimize the potential influence of confounding factors, we employed propensity score matching, inverse probability weighted (IPW) Cox proportional hazard regression, and multivariable Cox proportional hazard regression to adjust for differences in baseline characteristics. The propensity score was calculated through logistic regression taking into account clinical and lesion variables (age, sex, hypertension, diabetes mellitus, current smoking, previous MI, previous PCI, end-stage renal disease, clinical presentation status, left ventricular ejection fraction, left main disease, bifurcation lesion, and the number of diseased vessels). A 1:1 nearest neighbor matching without replacement was performed with a caliper size of 0.2 of the propensity score. Following propensity score matching or IPW adjustment, we evaluated the balance between the two groups by calculating absolute standardized mean differences. Successful balance between the comparative groups was considered as achieved when absolute standardized mean differences for all relevant covariates were 0.1 or less (Supplementary Table S1). 

Survival curves for 2-year MACEs were constructed using Kaplan–Meier estimates and compared using the log-rank test. Hazard ratios (HRs) with 95% confidence intervals (CIs) for the treatment groups as an independent predictor of MACEs were calculated through multivariable Cox regression analysis. The assumption of proportionality was assessed using two-sided *p*-values, and a value of <0.05 was considered statistically significant. Statistical analyses in this study were conducted using R version 4.3.2 (R Foundation for Statistical Computing, Vienna, Austria).

## 3. Results

From October 2010 to November 2022, 1388 patients underwent PCI based on DCB, of which 53 patients had coronary artery bypass surgery, while 1135 patients had no CTO lesions; therefore, 200 patients were included in the analysis (Figure S1). In the DES treatment group, a total of 13,160 patients were screened, with DES treated between July 2003 and August 2018, which was from the PTRG-DES consortium, of which 11,226 patients utilized second-generation DESs. Excluding 139 patients who had previously undergone coronary artery bypass graft surgery, we further excluded 10,329 patients without CTOs. Supplementary Figure S1 illustrates the study group diagram, indicating how the final population included in this study was identified.

The baseline clinical and lesion characteristics of the patients are summarized in Table 1 and Table 2. A total of 861 patients underwent PCI successfully for CTO lesions. The mean age of the patients was 63.3 ± 11.1 years, and the majority of patients were men. The two groups differed significantly in clinical and lesion characteristics across the overall population. The DCB treatment group had a higher proportion of men, previous PCI, and more frequent end-stage renal disease, presentations with unstable angina, left main disease, bifurcation disease, and target lesion of left circumflex artery. The DES-only group had a higher prevalence of dyslipidemia, current smokers, and acute MI presentation.

In the DCB-based group, 49.0% of the patients were treated with DCB alone and 51.0% were treated with the hybrid approach using DESs. For the devices used in the DCB-based group, 66.0% were DCBs and 34.0% were DESs. In the DCB-based group, bailout stenting was performed in seven patients (3.5%). The procedure characteristics are summarized in Table 2. The total number of diseased vessels, the total number of treated vessels, and the total number of used devices were higher in the DCB-based PCI group. The total device length was higher in the DCB-based group (60.0 mm; IQR: 36.5–86.0 mm in the DCB-based group; and 42.0 mm; IQR: 28.0–67.0 mm in the DES-only group, *p* < 0.001), but the mean device diameter was larger in the DES-only group (2.8 mm; IQR: 2.5–3.0 mm) compared to that of the DCB-based group (2.7 mm; IQR:2.5–2.9 mm) (*p* < 0.001). 

A statistically significant difference was observed in the number of DCBs and DESs used in both the groups. In the DCB-based group, the number of DCBs was 1.0 (IQR: 1.0–2.0) and that of DESs was 1.0 (IQR: 0.0–1.0). The total DCB length in the DCB-based group was 30.0 mm (IQR: 26.0–56.0 mm), and the mean DCB diameter was 2.5 mm (IQR: 2.5–2.7 mm). In the DCB-based group, a small DCB with a diameter of ≤2.5 mm was utilized in 64.5% of the patients. There were significant differences observed in the total lengths and mean diameters of the DESs used between the two groups. The total DES length was 6.5 mm (IQR: 0.0–38.0 mm) in the DCB-based group and 42.0 mm (IQR: 28.0–67.0 mm) in the DES-only group (*p* < 0.001). The mean DES diameter was larger in the DCB-based group and was 3.0 mm (IQR: 2.8–3.5 mm) compared to 2.8 mm (IQR: 2.5–3.0 mm) in the DES-only group (*p* < 0.001). A significant disparity between the two groups was evident when the DES diameter was ≤2.5 mm (9.8% in the DCB-based group vs. 36.5% in the DES-only group, *p* < 0.001). In comparison with the DES-only group, the DCB-based group saw a notable reduction in both the number of stents and the total stent length by 66.0% and 63.9%, respectively, in the DCB-based group compared with those of the DES-only group. 

### 3.1. Two-Year Outcomes

**Figure 1:**
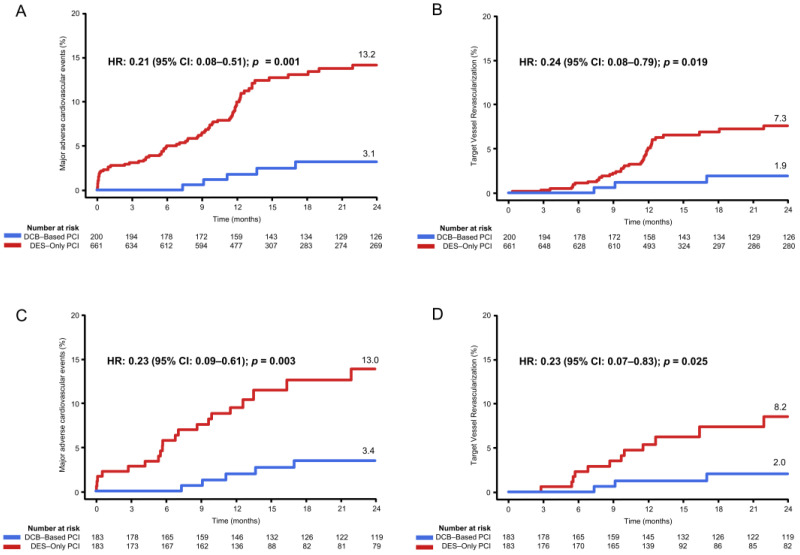
Cumulative incidence of major adverse cardiovascular events and target vessel revascularization in the overall population and propensity-matched population. (**A**) Major adverse cardiovascular events in overall population. (**B**) Target vessel revascularization in overall population. (**C**) Major adverse cardiovascular events in propensity-matched population. (**D**) Target vessel revascularization in propensity-matched population. DCB, drug-coated balloon; DES, drug-eluting stent; PCI, percutaneous coronary intervention; HR, hazard ratio; and CI, confidence interval.

**Figure 2:**
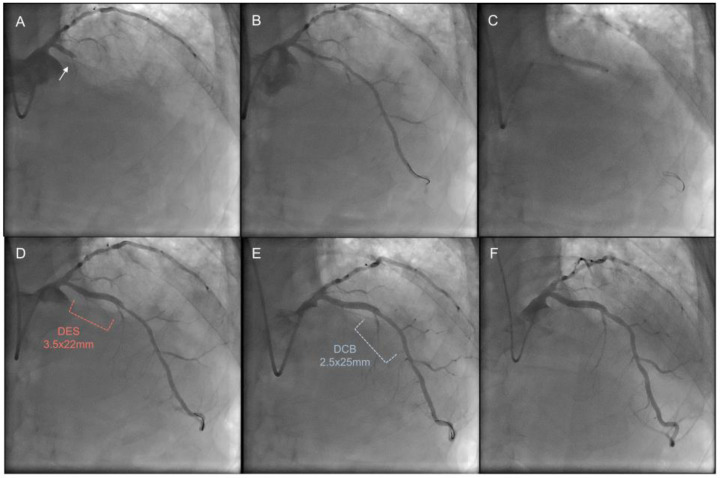
Representative case from the drug-coated balloon-based treatment group. (**A**) A CTO lesion in the LAD. The arrow points to the CTO entrance. (**B**) Angiogram after predilation. (**C**) A DES implantation at proximal LAD. (**D**) After DES implantation, diffuse stenotic lesion is seen in the middle of LAD. (**E**) It was treated with a small diameter DCB. (**F**) Follow-up angiogram after 6 months. CTO, chronic total occlusion; LAD, left anterior descending artery; DES, drug-eluting stent; and DCB, drug-coated balloon.

Clinical follow-up was available in all patients (100%). A comparison of clinical outcomes between the DCB-based PCI group and the DES-only PCI group is presented in Table 3 and Figure 1. The median follow-up duration was 1155.0 days (IQR: 424.5–2004.0 days) in the DCB-based group and 466.0 days (IQR: 365.0–1653.0 days) in the DES-only PCI group. The risk of MACEs was significantly lower in the DCB-based PCI group than that in the DES-only PCI group (3.1% vs. 13.2%; HR: 0.21; 95% CI: 0.08–0.51; *p* = 0.001). The risk of target vessel revascularization and major bleeding was also significantly lower in the DCB-based PCI group than that in the DES-only PCI group (1.9% vs. 7.3%; HR: 0.24; 95% CI: 0.08–0.79; *p* = 0.019). Major bleeding was higher in the DES-only group (0 vs. 4.6%; HR: 0.06; 95% CI: 0.00–0.41; *p* = 0.001). No target lesion thrombosis and major bleeding were observed in the DCB-based treatment group. Representative images are shown in Figure 2.

Across sensitivity analyses utilizing multivariable Cox regression, propensity score matching and IPW adjustment consistently lower the risks of MACEs in the DCB-based PCI group compared to those in the DES-only PCI group (Table 3).

### 3.2. Independent Predictors of Composite Outcomes

Multivariable Cox proportional hazard models identified the independent predictors of MACEs and target vessel revascularization (Supplementary Table S2). DCB-based PCI was independently associated with a decreased risk of MACE (HR: 0.21; 95% CI: 0.08 to 0.52; *p* = 0.001) and target vessel revascularization (HR: 0.25; 95% CI: 0.07 to 0.81; *p* = 0.021) at 2 years.

### 3.3. Subgroup Analysis

**Figure 3:**
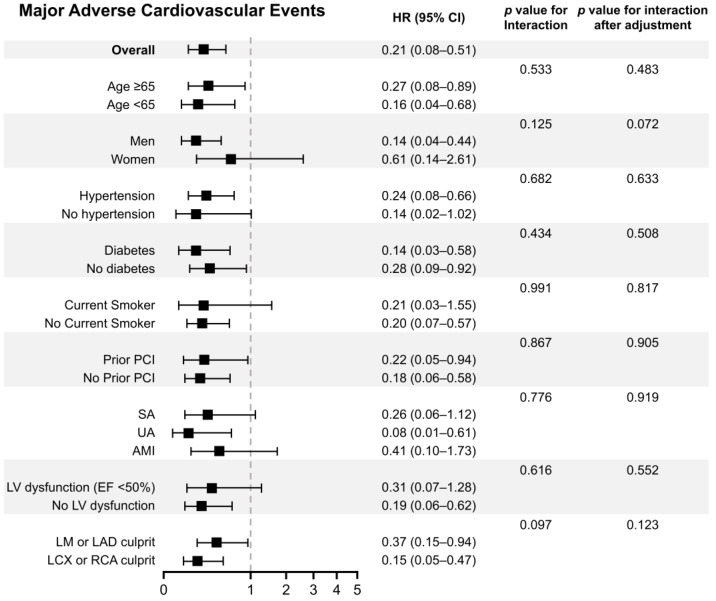
Exploratory subgroup analysis for major adverse cardiovascular events. PCI indicates percutaneous coronary intervention; SA, stable angina; UA, unstable angina; AMI, acute myocardial infarction; LV, left ventricular; EF, ejection fraction; LM, left main; LAD, left anterior descending artery; LCX, left circumflex artery; RCA, right coronary artery; DCB, drug-coated balloon; DES, drug-eluting stent; HR = hazard ratio; and CI = confidence interval.

Figure 3 presents the prognostic impact of DCB-based PCI among various subgroups. The significantly reduced risk of MACEs in the DCB-based PCI group compared to that in the DES-only PCI group remained consistent across all subgroups with no significant interaction *p* values.

## 4. Discussion

The main finding of the present study was that the DCB-based treatment approach for CTO CAD, either using DCB alone or in combination with DES guided by the International Consensus Group [18] and Asian Pacific Consensus Group [19], significantly and safely reduced the stent length and the number of stents. This reduction was particularly notable in terms of the small diameter of DES. This resulted in a lower risk of MACEs at 2 years compared to that of the DES-only treatment. Furthermore, sensitivity analyses using multivariable Cox regression, propensity score matching, and IPW adjustment consistently showed significantly lower risks of MACEs in the DCB-based PCI compared to those of the DES-only PCI group. Therefore, our data suggest that the DCB-based treatment approach is a reasonable strategy to improve outcomes in patients with CTO CAD.

In a society with an increasingly aging population, the issue of CTO CAD has become a challenge for interventional cardiologists. The CTO lesion is not rare, and a big registry reported that 16% of patients with CAD had CTO lesion [1]. Thus far, despite advancements in CTO recanalization techniques, operator expertise, a more standardized approach, and improved devices, including DES platforms, the results regarding the clinical advantages of CTO PCI over medication alone remains inconclusive [2,3,4,24]. Given the nature of the lesion, CTO often requires the use of a lengthy stent, similar to treating a diffuse long lesion. A long stent length is known to independently predict in-stent restenosis and stent thrombosis [6,7,8]. Recently, the GRAND-DES registry with the use of second-generation DESs demonstrated that long stent length (>40 mm) was significantly associated with higher target lesion failure and definite or probable stent thrombosis [9]. The study also demonstrated that long stent length is an independent predictor for ischemic clinical events in patients undergoing PCI [8]. Furthermore, performing a “full-metal jacket” PCI using overlapping DESs is associated with a high adverse event rate [10]. In a previous trial, two independent predictors of target lesion failure in “full-metal jacket” PCI were identified: number of target vessel DESs (hazard ratio: 1.72; 95% confidence interval: 1.16 to 2.54; *p* = 0.006) and persistent distal luminal narrowing (hazard ratio: 2.73, 95% confidence interval: 1.66 to 4.47; *p* < 0.001) [25]. Therefore, many CTO operators recommend avoiding the overtreatment of distal lesions with DESs because they often improve over time after the restoration of antegrade flow [26,27,28]. Additionally, stent implantation can hinder the restoration of vasomotion in stented segments and accelerate the development of neoatherosclerosis [11,12,13]. 

The use of modern, new-generation DESs has shown significant improvements in reducing adverse events compared to bare-metal stents and first-generation DESs. Nevertheless, contemporary DESs still pose risks of short- and long-term device-related adverse cardiovascular events due to their permanent metallic cage, which can increase the likelihood of stent thrombosis and in-stent restenosis [29,30,31]. CTO CAD, particularly involving complex and long lesions in small vessels, presents a challenging scenario in interventional cardiology with no unequivocal evidence on the best treatment approach. To address these concerns, efforts have been made to develop new treatment strategies, such as the hybrid approach involving a combination of DCBs and DESs. The hybrid approach offers the advantage of reducing the length of the permanent metallic cage, potentially leading to lower rates of stent thrombosis and restenosis. In this strategy, consecutive lesions are addressed individually, where a DES is placed in the proximal segment, including the CTO entrance, and a DCB treatment is performed for the distal lesion, which experiences negative remodeling due to the absence of antegrade flow for long time. Several observational studies have shown the feasibility and safety of this approach [32,33]. Similar to the previous study, this study also reduced the stent length, including the number of stents, in the DCB-based treatment group, with a particular focus on reducing the utilization of small stent diameters. This approach was employed for over half of the patients. In these cases, DESs were utilized in the larger, more proximal lesions, such as CTO entrance sites, while DCBs were reserved for smaller, more distal coronary lesions. This hybrid approach offers several advantages, including minimizing the overall stent burden, especially in distal lesions with smaller diameters, and treating the underlying disease while maintaining access for potential future coronary artery bypass grafting, if necessary. Decreasing metal stent length may also help maintain the vessel’s response to vasomotion stimuli and reduce the risk of neoatherosclerosis [17]. While the development of devices, procedural techniques, and medication regimens to prevent very late events is crucial for improving lifelong outcomes in patients undergoing coronary revascularization, adopting a DCB-based treatment approach offers potential benefits for managing CTO lesions. Further studies are needed for a comprehensive evaluation of the role of DCB in this context.

### Limitations

Our study has some limitations. Firstly, it is crucial to acknowledge that this study possesses inherent limitations stemming from its observational nature and reliance on registry data. Furthermore, the allowance for physicians to opt for the treatment strategy introduces the potential for selection bias. To mitigate this bias, extensive sensitivity analyses were undertaken, adjusting for both measured and unmeasured confounders to minimize the impact of varying baseline characteristics. Secondly, the study population was drawn from an expert center specialized in exclusive DCB treatment for de novo CAD. Consequently, the findings might not be easily replicable without a substantial learning curve and proficiency in this particular treatment approach. Thirdly, discrepancies in the results might arise due to disparities in the enrollment periods of the two groups. While the PTRG-DES registry has been operational since 2003, the patients included in the propensity match analysis were instances involving second-generation DESs. Hence, significant differences stemming from device development and enhancements in PCI techniques between the two groups are not anticipated. For more robust and comprehensive insights into the long-term outcomes of DCB-based treatment in CTO lesion PCI, further prospective randomized non-inferiority or superiority clinical trials with more extensive patient cohorts are imperative.

## 5. Conclusions

Despite improvements in modern DESs, concerns remain about negative effects related to stents, especially for patients with CTO CAD. Using the DCB-based treatment approach, either with DCB alone or combined with DESs, has shown great potential in reducing the stent-related adverse events, particularly by reducing the use of smaller DESs. Additionally, this approach led to a lower risk of MACEs one year after treatment compared to that when only DESs were used. Our study adds to the growing evidence that supports the DCB-based approach as a promising strategy for effectively managing this difficult condition. To further confirm the safety and effectiveness of the DCB-based treatment in CTO PCI, future research, including larger randomized controlled trials, will be crucial.

## Figures and Tables

**Table 1:** Baseline clinical characteristics in the overall population and the propensity-matched population.

	Overall (*n* = 861)	Overall Population			Propensity-Matched Population		
		DCB-Based (*n* = 200)	DES-Only (*n* = 661)	*p* Value	DCB-Based (*n* = 183)	DES-Only (*n* = 183)	*p* Value
Age, years	63.3 ± 11.1	62.2 ± 10.6	63.7 ± 11.3	0.108	62.5 ± 10.6	62.9 ± 10.4	0.754
Men	632 (73.4)	168 (84.0)	464 (70.2)	<0.001	153 (83.6)	155 (84.7)	0.886
Hypertension	543 (63.1)	138 (69.0)	405 (61.3)	0.057	123 (67.2)	123 (67.2)	>0.999
Diabetes	343 (39.8)	89 (44.5)	254 (38.4)	0.146	81 (44.3)	83 (45.4)	0.916
Dyslipidemia	577 (67.0)	120 (60.0)	457 (69.1)	0.020	111 (60.7)	121 (66.1)	0.329
Current smoker	246 (28.6)	45 (22.5)	201 (30.4)	0.038	43 (23.5)	37 (20.2)	0.527
Prior MI	89 (10.3)	25 (12.5)	64 (9.7)	0.310	22 (12.0)	21 (11.5)	>0.999
Prior PCI	146 (17.0)	45 (22.5)	101 (15.3)	0.023	40 (21.9)	40 (21.9)	>0.999
End-stage renal disease	17 (2.0)	10 (5.0)	7 (1.1)	0.001	6 (3.3)	5 (2.7)	>0.999
Clinical presentation							
Stable angina	374 (43.4)	81 (40.5)	293 (44.3)	0.381	71 (38.8)	69 (37.7)	0.914
Unstable angina	272 (31.6)	83 (41.5)	189 (28.6)	0.001	76 (41.5)	80 (43.7)	0.751
Acute MI	215 (25.0)	36 (18.0)	179 (27.1)	0.012	36 (19.7)	34 (18.6)	0.894
Left ventricular ejection fraction, %	55.78 ± 11.11	56.8 ± 9.9	55.5 ± 11.4	0.114	56.8 ± 9.9	57.4 ± 11.5	0.587

Values are mean ± SD or *n* (%). MI, myocardial infarction; PCI, percutaneous coronary intervention; DCB, drug-coated balloon; and DES, drug-eluting stent.

**Table 2:** Baseline lesion- and procedure-related profiles.

	Overall Population			Propensity-Matched Population		
	DCB-Based (*n* = 200)	DES-Only (*n* = 661)	*p* Value	DCB-Based (*n* = 183)	DES-Only (*n* = 183)	*p* Value
DCB-only treatment	98 (49.0)	0		92 (50.3)	0	
Location of target lesion						
Left main	24 (12.0)	19 (2.9)	<0.001	18 (9.8)	14 (7.7)	0.579
Bifurcation	69 (34.5)	100 (15.1)	<0.001	57 (31.1)	52 (28.4)	0.648
LAD	117 (58.5)	371 (56.1)	0.609	102 (55.7)	123 (67.2)	0.032
LCX	112 (56.0)	228 (34.5)	<0.001	99 (54.1)	91 (49.7)	0.464
RCA	110 (55.0)	348 (52.6)	0.615	100 (54.6)	99 (54.1)	>0.999
Total number of diseased vessels	2.0 (2.0–3.0)	1.0 (1.0–2.0)	<0.001	2.0 (2.0–3.0)	2.0 (1.0–3.0)	0.691
Total number of treated vessels	2.0 (1.0–2.0)	1.0 (1.0–2.0)	<0.001	2.0 (1.0–2.0)	2.0 (1.0–2.0)	0.199
Procedural characteristics						
Total number of used devices	2.0 (2.0–3.0)	2.0 (1.0–3.0)	<0.001	2.0 (2.0–3.0)	2.0 (1.0–3.0)	0.385
Total device length, mm	60.0 (36.5–86.0)	42.0 (28.0–67.0)	<0.001	60.0 (35.0–85.0)	51.0 (30.0–73.0)	0.007
Mean device diameter, mm	2.7 (2.5–2.9)	2.8 (2.5–3.0)	<0.001	2.7 (2.5–2.9)	2.8 (2.5–3.0)	0.144
Total number of used DCBs	1.0 (1.0–2.0)	0		1.0 (1.0–2.0)	0	
Total DCB length, mm	30.0 (26.0–56.0)	0		30.0 (26.0–56.0)	0	
Mean DCB diameter, mm	2.5 (2.5–2.7)	0		2.5 (2.5–2.7)	0	
Small DCBs used (≦2.5 mm)	129 (64.5)	0		118 (64.5)	0	
Total number of used DESs	1.0 (0.0–1.0)	2.0 (1.0–3.0)	<0.001	0.0 (0.0–1.0)	2.0 (1.0–3.0)	<0.001
Total DES length, mm	6.5 (0.0–38.0)	42.0 (28.0–67.0)	<0.001	0.0 (0.0–38.0)	51.0 (30.0–73.0)	<0.001
Mean DES diameter, mm	3.0 (2.8–3.5)	2.8 (2.5–3.0)	<0.001	3.0 (2.8–3.5)	2.8 (2.5–3.0)	<0.001
Small DESs used (≦2.5 mm)	10/102 (9.8)	241/661 (36.5)	<0.001	9/91 (9.9)	76/183 (41.5)	<0.001

Values are *n* (%) or median (quartile 1–quartile 3). LAD, left anterior descending artery; LCX, left circumflex artery; RCA, right coronary artery; DCB, drug-coated balloon; DES, drug-eluting stent; and PCI, percutaneous coronary intervention.

**Table 3:** Comparison of 2-year clinical outcomes according to treatment strategy.

	DCB-Based PCI (*n* = 200)	DES-Only PCI (*n* = 661)	Unadjusted		Multivariable-Adjusted		Propensity Score-Matched		IPW-Adjusted	
			HR (95% CI)	*p* Value	HR (95% CI)	*p* Value	HR (95% CI)	*p* Value	HR (95% CI)	*p* Value
Major adverse cardiovascular events	5 (3.1)	74 (13.2)	0.21 (0.08–0.51)	0.001	0.17 (0.06–0.43)	<0.001	0.23 (0.09–0.61)	0.003	0.28 (0.11–0.71)	0.008
Cardiac death	2 (1.3)	12 (2.3)	0.51 (0.11–2.30)	0.384	0.48 (0.09–2.51)	0.384	0.99 (0.15–6.69)	0.988	0.56 (0.12–2.80)	0.507
Myocardial infarction	1 (0.8)	4 (0.7)	0.76 (0.08–6.86)	0.808	0.32 (0.01–7.07)	0.470	0.47 (0.05–4.27)	0.499	1.61 (0.21–12.1)	0.643
Stent or target lesion thrombosis	0	2 (0.3)	0.66 (0.00–8.17)	0.783	0.55 (0.00–8.17)	0.708	-	-	-	-
Target vessel revascularization	3 (1.9)	39 (7.3)	0.24 (0.08–0.79)	0.019	0.23 (0.07–0.80)	0.021	0.23 (0.07–0.83)	0.025	0.35 (0.10–1.20)	0.094
Major bleeding	0	27 (4.6)	0.06 (0.00–0.41)	0.001	0.05 (0.00–0.39)	0.001	0.07 (0.00–0.60)	0.011	-	-

Values are *n* (%) unless otherwise indicated. The cumulative incidences of clinical outcomes are presented as Kaplan–Meier estimates. Multivariable Cox proportional hazard regression model, propensity score-matched cohort, and inverse probability of treatment weighting method were used to adjust for baseline differences between comparative groups. Major adverse cardiovascular events were composed of cardiac death, myocardial infarction, stent or target lesion thrombosis, target vessel revascularization, and major bleeding (Bleeding Academic Research Consortium bleeding type 3 or greater). DCB, drug-coated balloon; DES, drug-eluting stent; PCI, percutaneous coronary intervention; HR, hazard ratio; CI, confidence interval; and IPW, inverse probability weighted.

## Data Availability

The data presented in this study are available on request from the corresponding authors.

## Supplementary Materials

The following supporting information can be downloaded at: https://www.mdpi.com/article/10.3390/jcm13123381/s1, Table S1. Absolute standardized differences of variables among unadjusted, propensity-score matched, and IPW-adjusted cohort. Table S2. Independent predictors for major adverse cardiovascular events or target vessel revascularization. Figure S1. Study group diagram indicating how the final population included in this study was identified. DCB indicates drug-coated balloon; PCI, percutaneous coronary intervention; CABG, coronary artery bypass graft; CTO, chronic total occlusion; DES, drug-eluting stent; LVEF, left ventricular ejection fraction.

## References

[1] Råmunddal T., Hoebers L.P., Henriques J.P., Dworeck C., Angerås O., Odenstedt J., Ioanes D., Olivecrona G., Harnek J., Jensen U. (2014). Chronic total occlusions in Sweden—A report from the Swedish Coronary Angiography and Angioplasty Registry (SCAAR). PLoS ONE.

[2] Lee P.H., Lee S.W., Park H.S., Kang S.H., Bae B.J., Chang M., Roh J.H., Yoon S.H., Ahn J.M., Park D.W. (2016). Successful recanalization of native coronary chronic total occlusion is not associated with improved long-term survival. JACC Cardiovasc. Interv..

[3] Henriques J.P., Hoebers L.P., Råmunddal T., Laanmets P., Eriksen E., Bax M., Ioanes D., Suttorp M.J., Strauss B.H., Barbato E. (2016). Percutaneous intervention for concurrent chronic total occlusions in patients with STEMI: The EXPLORE trial. J. Am. Coll. Cardiol..

[4] Lee S.W., Lee P.H., Ahn J.M., Park D.W., Yun S.C., Han S., Kang H., Kang S.J., Kim Y.H., Lee C.W. (2019). Randomized trial evaluating percutaneous coronary intervention for the treatment of chronic total occlusion. Circulation.

[5] Obedinskiy A.A., Kretov E.I., Boukhris M., Kurbatov V.P., Osiev A.G., Ibn Elhadj Z., Obedinskaya N.R., Kasbaoui S., Grazhdankin I.O., Prokhorikhin A.A. (2018). The IMPACTOR-CTO trial. JACC Cardiovasc. Interv..

[6] Lee C.W., Park D.W., Lee B.K., Kim Y.H., Hong M.K., Kim J.J., Park S.W., Park S.J. (2006). Predictors of restenosis after placement of drug-eluting stents in one or more coronary arteries. Am. J. Cardiol..

[7] D’Ascenzo F., Bollati M., Clementi F., Castagno D., Lagerqvist B., de la Torre Hernandez J.M., ten Berg J.M., Brodie B.R., Urban P., Jensen L.O. (2013). Incidence and predictors of coronary stent thrombosis: Evidence from an international collaborative meta-analysis including 30 studies, 221,066 patients, and 4276 thromboses. Int. J. Cardiol..

[8] Kang J., Park K.W., Lee H.S., Zheng C., Rhee T.M., Ki Y.J., Chang M., Han J.K., Yang H.M., Kang H.J. (2021). Relative impact of clinical risk versus procedural risk on clinical outcomes after percutaneous coronary intervention. Circ. Cardiovasc. Interv..

[9] Kong M.G., Han J.K., Kang J.H., Zheng C., Yang H.M., Park K.W., Kang H.J., Koo B.K., Chae I.H., Kim H.S. (2021). Clinical outcomes of long stenting in the drug-eluting stent era: Patient-level pooled analysis from the GRAND-DES registry. EuroIntervention.

[10] Sharp A.S., Latib A., Ielasi A., Larosa C., Godino C., Saolini M., Magni V., Gerber R.T., Montorfano M., Carlino M. (2009). Long-term follow-up on a large cohort of “full-metal jacket” percutaneous coronary intervention procedures. Circ. Cardiovasc. Interv..

[11] Yahagi K., Kolodgie F.D., Otsuka F., Finn A.V., Davis H.R., Joner M., Virmani R. (2016). Pathophysiology of native coronary, vein graft, and in-stent atherosclerosis. Nat. Rev. Cardiol..

[12] Togni M., Windecker S., Cocchia R., Wenaweser P., Cook S., Billinger M., Meier B., Hess O.M. (2005). Sirolimus-eluting stents associated with paradoxic coronary vasoconstriction. J. Am. Coll. Cardiol..

[13] Galassi A.R., Tomasello S.D., Crea F., Costanzo L., Campisano M.B., Marzá F., Tamburino C. (2012). Transient impairment of vasomotion function after successful chronic total occlusion recanalization. J. Am. Coll. Cardiol..

[14] Scheller B., Hehrlein C., Bocksch W., Rutsch W., Haghi D., Dietz U., Böhm M., Speck U. (2006). Treatment of coronary in-stent restenosis with a paclitaxel-coated balloon catheter. N. Engl. J. Med..

[15] Jeger R.V., Farah A., Ohlow M.A., Mangner N., Möbius-Winkler S., Weilenmann D., Wöhrle J., Stachel G., Markovic S., Leibundgut G. (2020). Long-term efficacy and safety of drug-coated balloons versus drug-eluting stents for small coronary artery disease (BASKET-SMALL 2): 3-year follow-up of a randomised, non-inferiority trial. Lancet.

[16] Jeger R.V., Farah A., Ohlow M.A., Mangner N., Möbius-Winkler S., Leibundgut G., Weilenmann D., Wöhrle J., Richter S., Schreiber M. (2018). Drug-coated balloons for small coronary artery disease (BASKET-SMALL 2): An open-label randomised non-inferiority trial. Lancet.

[17] Kim S., Lee J.S., Kim Y.H., Kim J.S., Lim S.Y., Kim S.H., Kim M., Ahn J.C., Song W.H. (2022). Favorable vasomotor function after drug-coated balloon-only angioplasty of de novo native coronary artery lesions. J. Clin. Med..

[18] Jeger R.V., Eccleshall S., Wan Ahmad W.A., Ge J., Poerner T.C., Shin E.S., Alfonso F., Latib A., Ong P.J., Rissanen T.T. (2020). Drug-coated balloons for coronary artery disease: Third report of the international DCB Consensus Group. JACC Cardiovasc. Interv..

[19] Her A.Y., Shin E.S., Bang L.H., Nuruddin A.A., Tang Q., Hsieh I.C., Hsu J.C., Kiam O.T., Qiu C., Qian J. (2021). Drug-coated balloon treatment in coronary artery disease: Recommendations from an Asia-Pacific Consensus Group. Cardiol. J..

[20] Shin E.S., Jun E.J., Kim S., Kim B., Kim T.H., Sohn C.B., Her A.Y., Park Y., Cho J.R., Jeong Y.H. (2023). Clinical impact of drug-coated balloon-based percutaneous coronary intervention in patients with multivessel coronary artery disease. JACC Cardiovasc. Interv..

[21] Her A.Y., Jeong Y.H., Kim B.K., Joo H.J., Chang K., Park Y., Song Y.B., Ahn S.G., Suh J.W., Lee S.Y. (2022). Platelet function and genotype after des implantation in East Asian patients: Rationale and characteristics of the PTRG-DES Consortium. Yonsei Med. J..

[22] Cutlip D.E., Windecker S., Mehran R., Boam A., Cohen D.J., van Es G.A., Steg P.G., Morel M.A., Mauri L., Vranckx P. (2007). Clinical end points in coronary stent trials: A case for standardized definitions. Circulation.

[23] Mehran R., Rao S.V., Bhatt D.L., Gibson C.M., Caixeta A., Eikelboom J., Kaul S., Wiviott S.D., Menon V., Nikolsky E. (2011). Standardized bleeding definitions for cardiovascular clinical trials: A consensus report from the Bleeding Academic Research Consortium. Circulation.

[24] Werner G.S., Hildick-Smith D., Martin Yuste V., Boudou N., Sianos G., Gelev V., Rumoroso J.R., Erglis A., Christiansen E.H., Escaned J. (2023). Three-year outcomes of a randomized multicentre trial comparing revascularization and optimal medical therapy for Chronic Total Coronary Occlusions (EuroCTO). EuroIntervention.

[25] Lee P.H., Lee S.W., Yun S.C., Bae J., Ahn J.M., Park D.W., Kang S.J., Kim Y.H., Lee C.W., Park S.W. (2017). Full metal jacket with drug-eluting stents for coronary chronic total occlusion. JACC Cardiovasc. Interv..

[26] Gomez-Lara J., Teruel L., Homs S., Ferreiro J.L., Romaguera R., Roura G., Sánchez-Elvira G., Jara F., Brugaletta S., Gomez-Hospital J.A. (2014). Lumen enlargement of the coronary segments located distal to chronic total occlusions successfully treated with drug-eluting stents at follow-up. EuroIntervention.

[27] Gasparini G.L., Rossi M.L., Presbitero P. (2014). Follow-up improvement of distal vessel diameter after successful chronic total coronary occlusion recanalization. JACC Cardiovasc. Interv..

[28] Kwon O., Lee P.H., Lee S.W., Kweon J., Lee J.Y., Lee K., Kang D.Y., Ahn J.M., Park D.W., Kang S.J. (2021). Fate of lumen size in distal coronary segment following successful chronic total occlusion recanalization. J. Cardiol..

[29] Madhavan M.V., Kirtane A.J., Redfors B., Généreux P., Ben-Yehuda O., Palmerini T., Benedetto U., Biondi-Zoccai G., Smits P.C., von Birgelen C. (2020). Stent-related adverse events >1 year after percutaneous coronary intervention. J. Am. Coll. Cardiol..

[30] Kufner S., Ernst M., Cassese S., Joner M., Mayer K., Colleran R., Koppara T., Xhepa E., Koch T., Wiebe J. (2020). 10-year outcomes from a randomized trial of polymer-free versus durable polymer drug-eluting coronary stents. J. Am. Coll. Cardiol..

[31] Madhavan M.V., Redfors B., Ali Z.A., Prasad M., Shahim B., Smits P.C., von Birgelen C., Zhang Z., Mehran R., Serruys P.W. (2020). Long-term outcomes after revascularization for stable ischemic heart disease: An individual patient-level pooled analysis of 19 randomized coronary stent trials. Circ. Cardiovasc. Interv..

[32] Jun E.J., Shin E.S., Teoh E.V., Bhak Y., Yuan S.L., Chu C.M., Garg S., Liew H.B. (2022). Clinical outcomes of drug-coated balloon treatment after successful revascularization of de novo chronic total occlusions. Front. Cardiovasc. Med..

[33] Köln P.J., Scheller B., Liew H.B., Rissanen T.T., Ahmad W.A., Weser R., Hauschild T., Nuruddin A.A., Clever Y.P., Ho H.H. (2016). Treatment of chronic total occlusions in native coronary arteries by drug-coated balloons without stenting—A feasibility and safety study. Int. J. Cardiol..

