# Supplementary material for: Impact of Drug-Coated Balloon-Based Revascularization in Patients with Chronic Total Occlusions
Source: J Clin Med. 2024 Jun 9;13(12):3381. doi: 10.3390/jcm13123381 (PMC11204241; doi:10.3390/jcm13123381)
Supplement: Supplementary file 1 [file jcm-13-03381-s001.zip › Supplementary Tables.pdf]

Supplementary table 1. Absolute standardized differences of variables among unadjusted, propensity-score matched, and IPW-adjusted cohort

	Unadjusted (n = 861)	propensity-score matched (n = 366)	IPW-adjusted (n = 861)
Age, years	0.132	0.033	0.048
Men	0.333	0.030	0.135
Hypertension	0.163	0.000	0.014
Diabetes mellitus	0.124	0.022	0.081
Dyslipidemia	0.192	0.114	0.198
Current smoker	0.180	0.079	0.040
Prior MI	0.090	0.017	0.038
Prior PCI	0.185	0.000	0.093
End-stage renal disease	0.231	0.032	0.077
Clinical presentation			
Stable angina	0.077	0.022	0.018
Unstable angina	0.273	0.044	0.102
Acute MI	0.219	0.028	0.093
Left ventricular ejection fraction, %	0.123	0.057	0.109
Location of target lesion			
Left main	0.353	0.077	0.118
Bifurcation	0.460	0.060	0.135
LAD	0.048	0.237	0.106
LCX	0.443	0.088	0.244
RCA	0.047	0.011	0.039
Total number of diseased vessel	0.718	0.049	0.249
Total number of treated vessel	0.413	0.156	0.054

MI, myocardial infarction; PCI, percutaneous coronary intervention; LAD, left anterior descending artery; LCX, left circumflex artery; RCA, right coronary artery; IPW, inverse probability weighting.

Supplementary table 2. Independent predictors for major adverse cardiovascular events or target vessel revascularization

	Hazard ratio	95% CI	*P* value
**Major adverse cardiovascular events**			
DCB-based PCI	0.21	0.08-0.52	0.001
Age >65	1.37	0.87-2.16	0.174
Men	1.31	0.78-2.19	0.307
Hypertension	1.09	0.67-1.77	0.727
Diabetes mellitus	1.66	1.05-2.63	0.030
Prior MI	1.72	0.92-3.19	0.088
End-stage renal disease	-	-	-
AMI presentation	1.33	0.81-2.18	0.253
LM or LAD target lesion	0.92	0.59-1.46	0.736
3-vessel disease	0.89	0.49-1.61	0.704
**Target vessel revascularization**			
DCB-based PCI	0.25	0.07-0.81	0.021
Age >65	1.00	0.54-1.87	0.989
Men	1.32	0.64-2.75	0.456
Hypertension	0.79	0.41-1.50	0.468
Diabetes mellitus	1.79	0.95-3.38	0.071
Prior MI	0.92	0.32-2.59	0.868
End-stage renal disease	-	-	-
AMI presentation	1.06	0.52-2.13	0.879
LM or LAD target lesion	0.97	0.52-1.80	0.915
3-vessel disease	0.80	0.34-1.86	0.602

Hazard ratios and their 95% confidence intervals were calculated by multivariable Cox regression analysis. Major adverse cardiovascular events were composed of cardiac death, myocardial infarction, stent or target lesion thrombosis, target vessel revascularization, and major bleeding (Bleeding Academic Research Consortium bleeding type 3 or greater). DCB, drug-coated balloon; PCI, percutaneous coronary intervention; AMI, acute myocardial infarction; LM: left main; LAD, left anterior descending artery; CI, confidence interval.
