# Supplementary figures and images for: Impact of Drug-Coated Balloon-Based Revascularization in Patients with Chronic Total Occlusions
Source: J Clin Med. 2024 Jun 9;13(12):3381. doi: 10.3390/jcm13123381 (PMC11204241; doi:10.3390/jcm13123381)

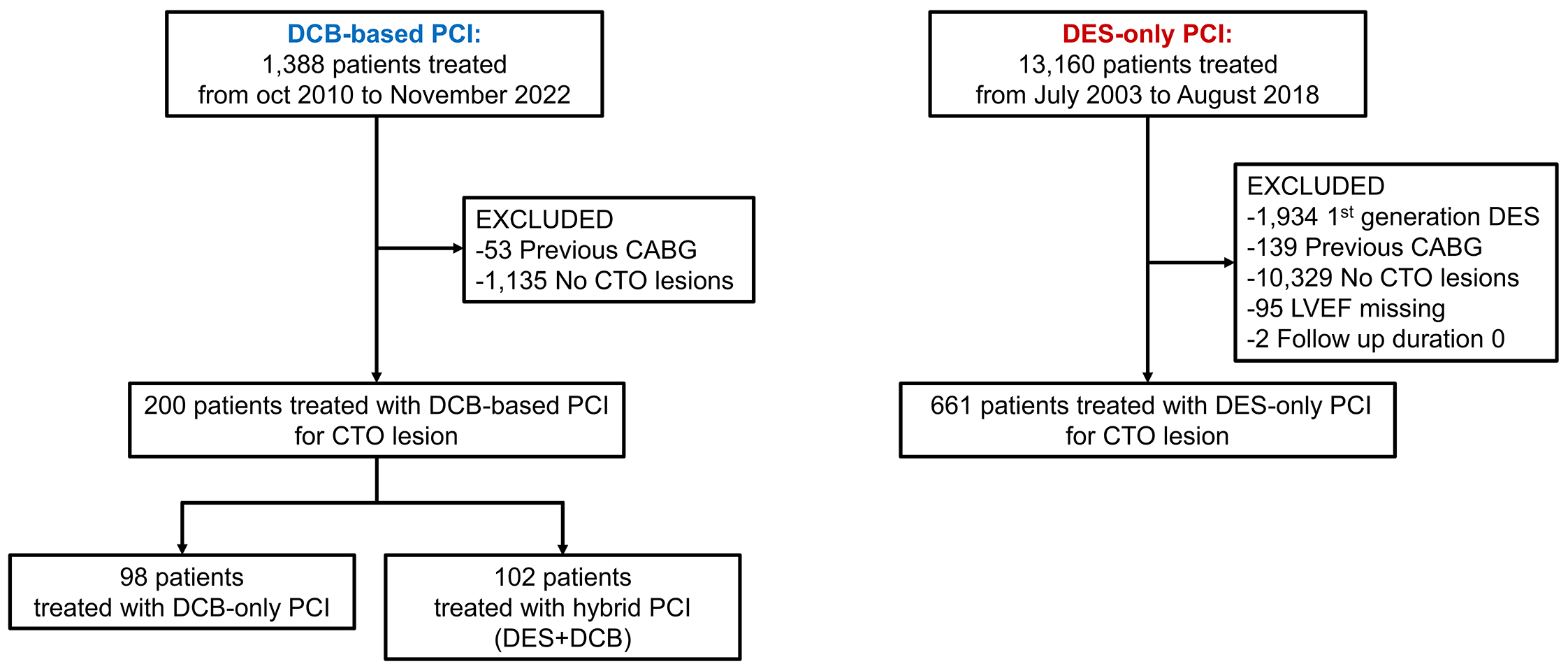

Supplement: Supplementary file 1 [file jcm-13-03381-s001.zip › Supplementary Figure 1.tif]
